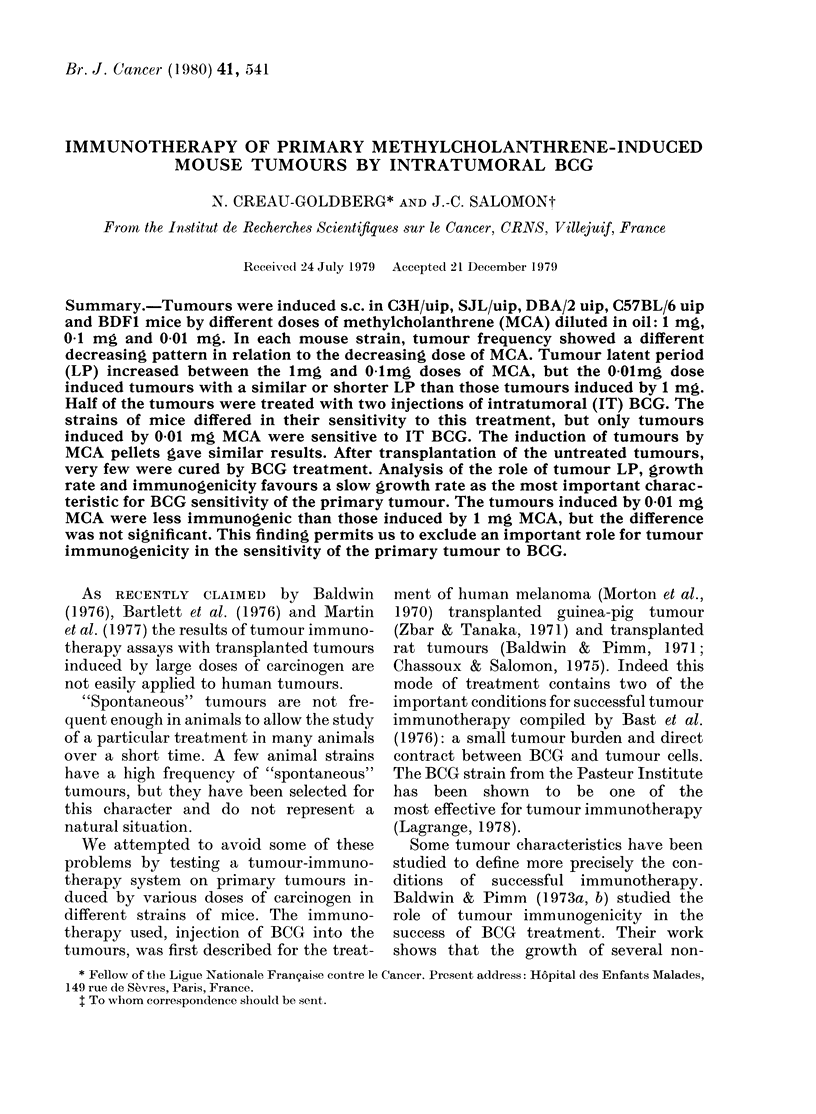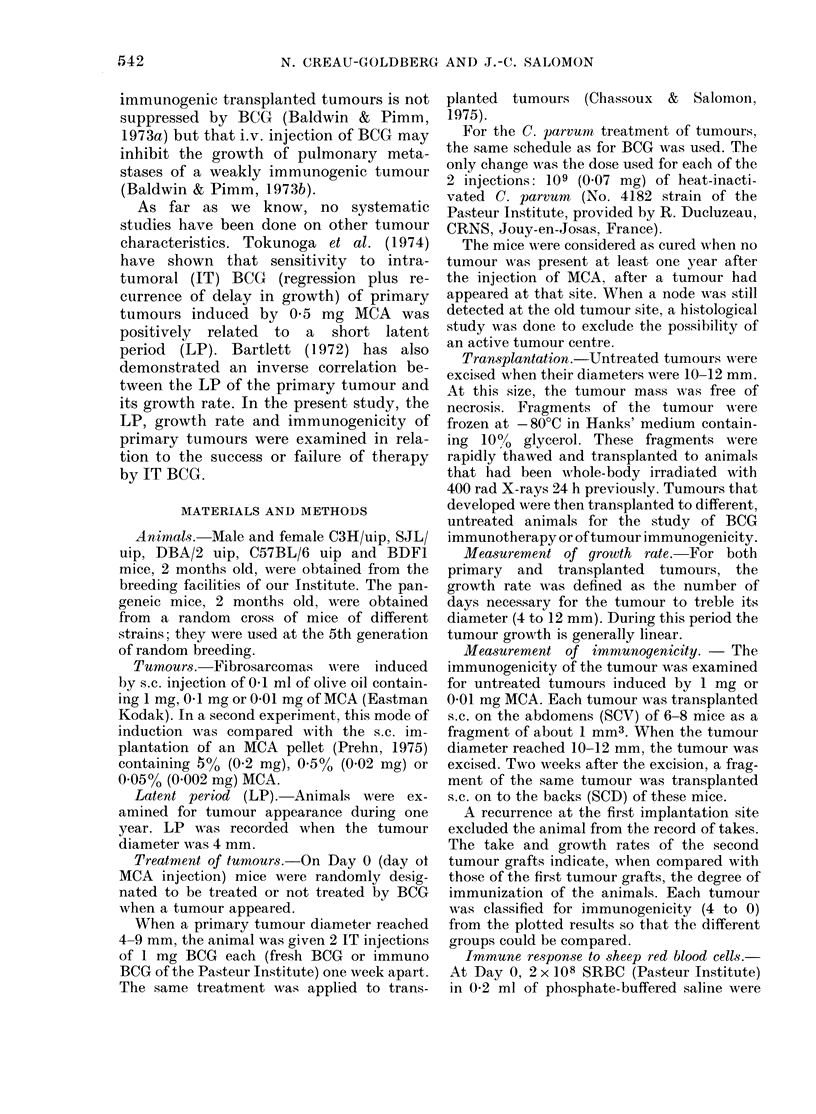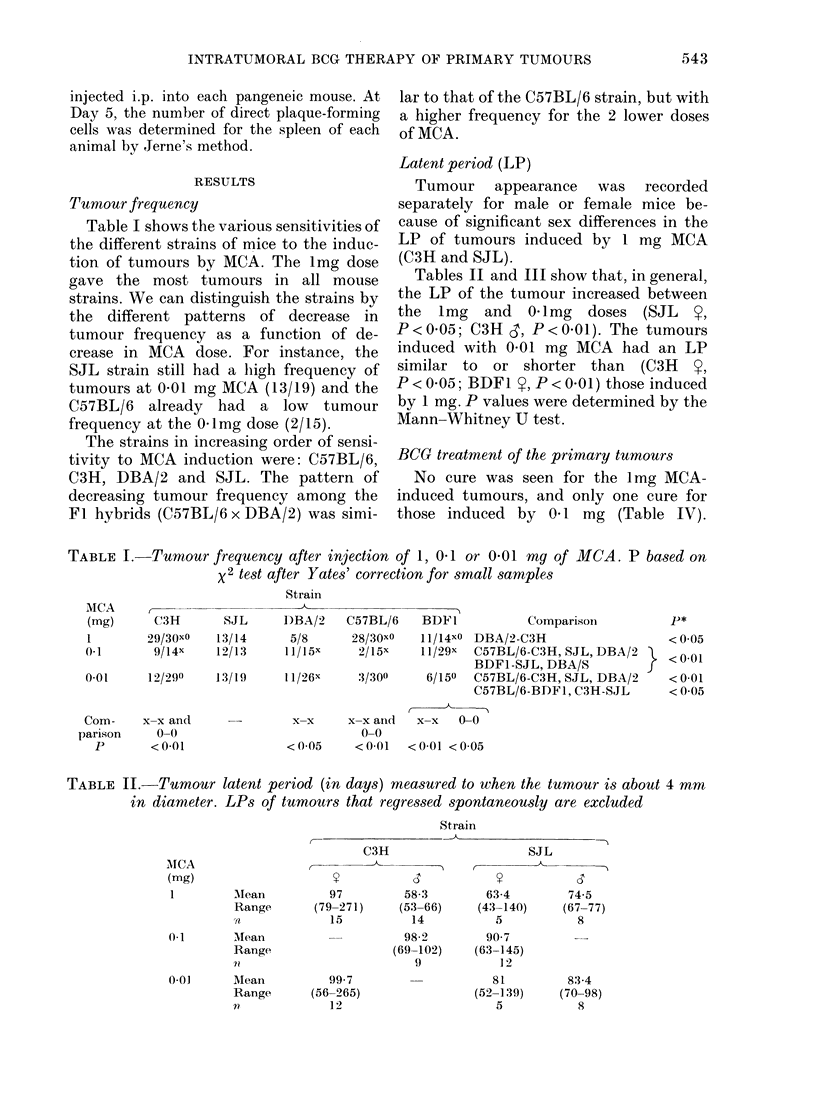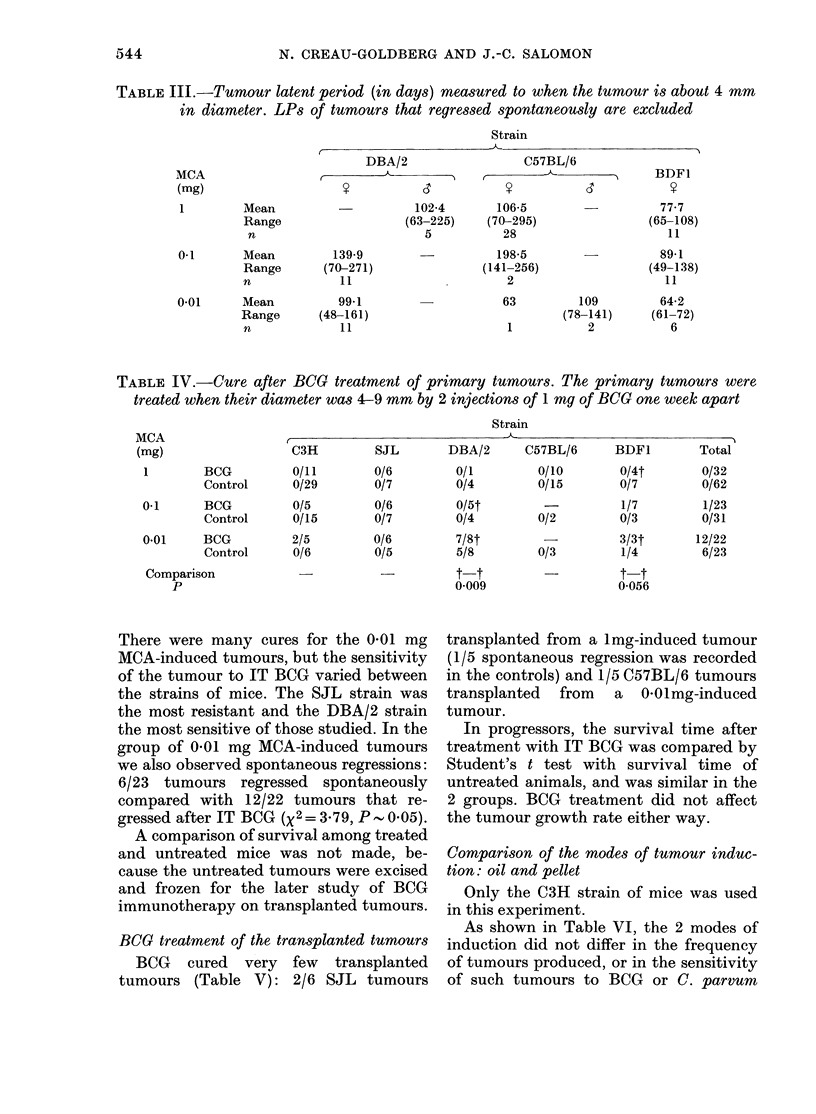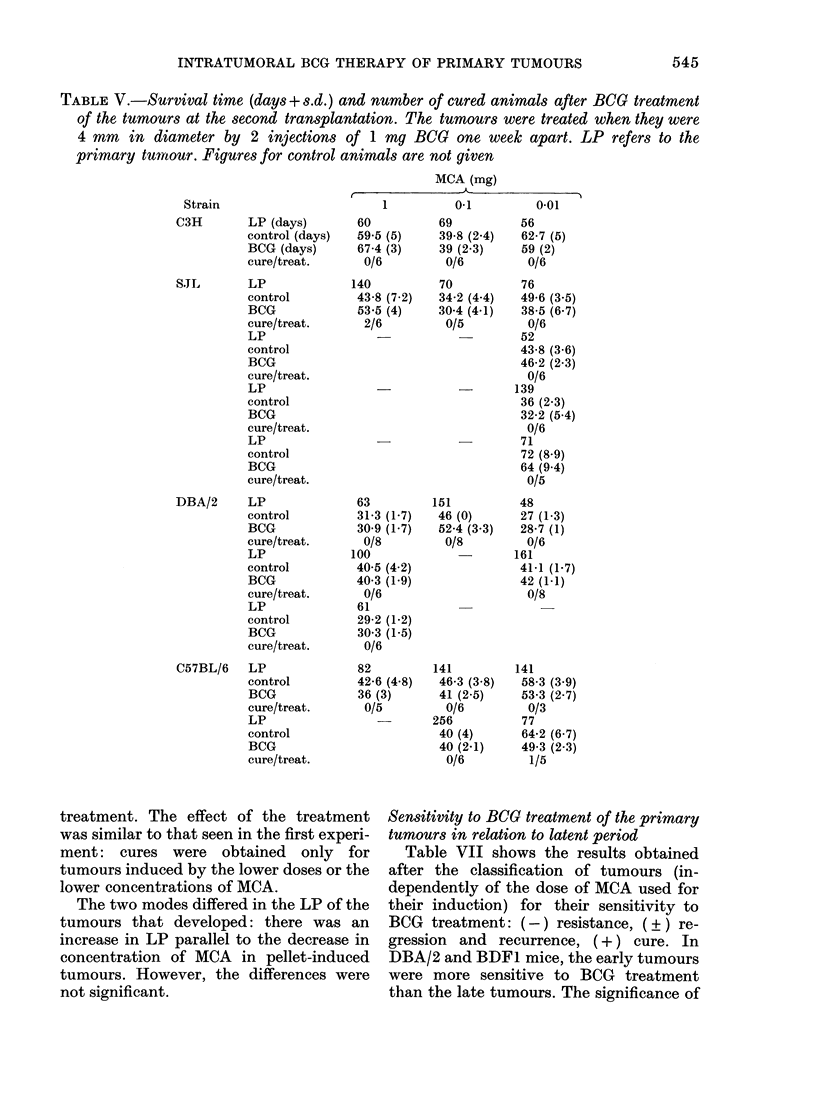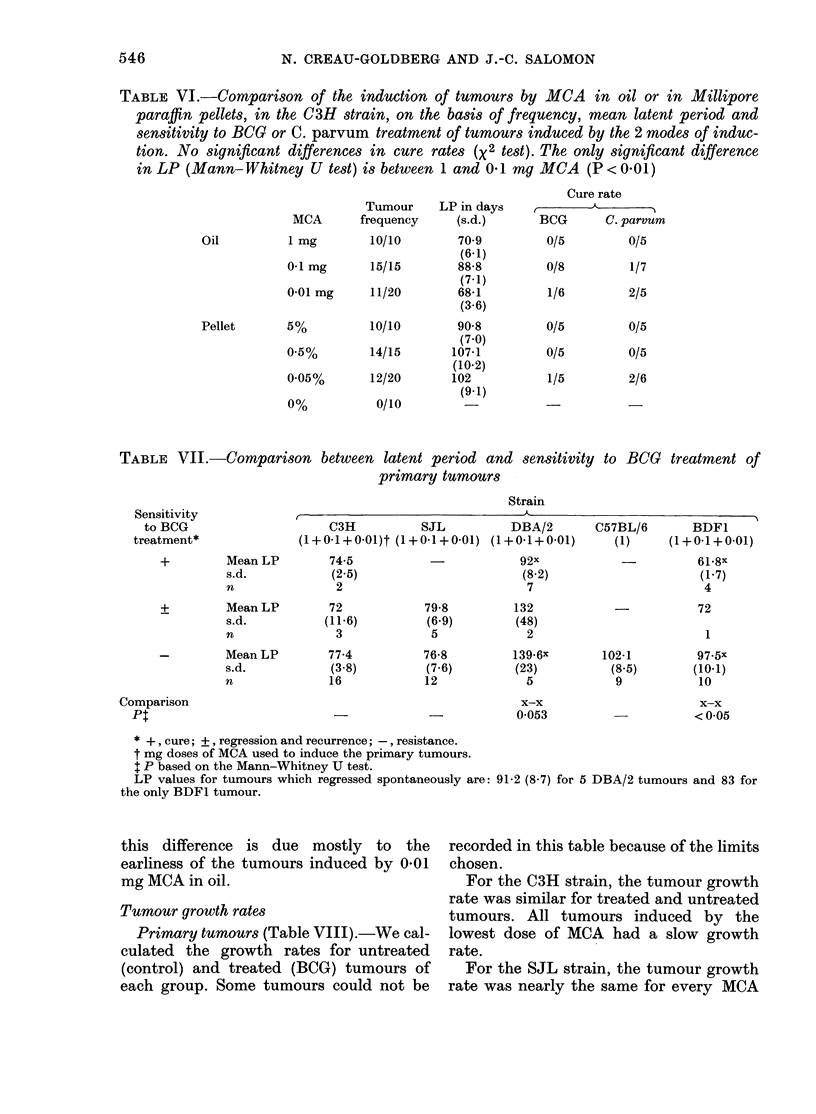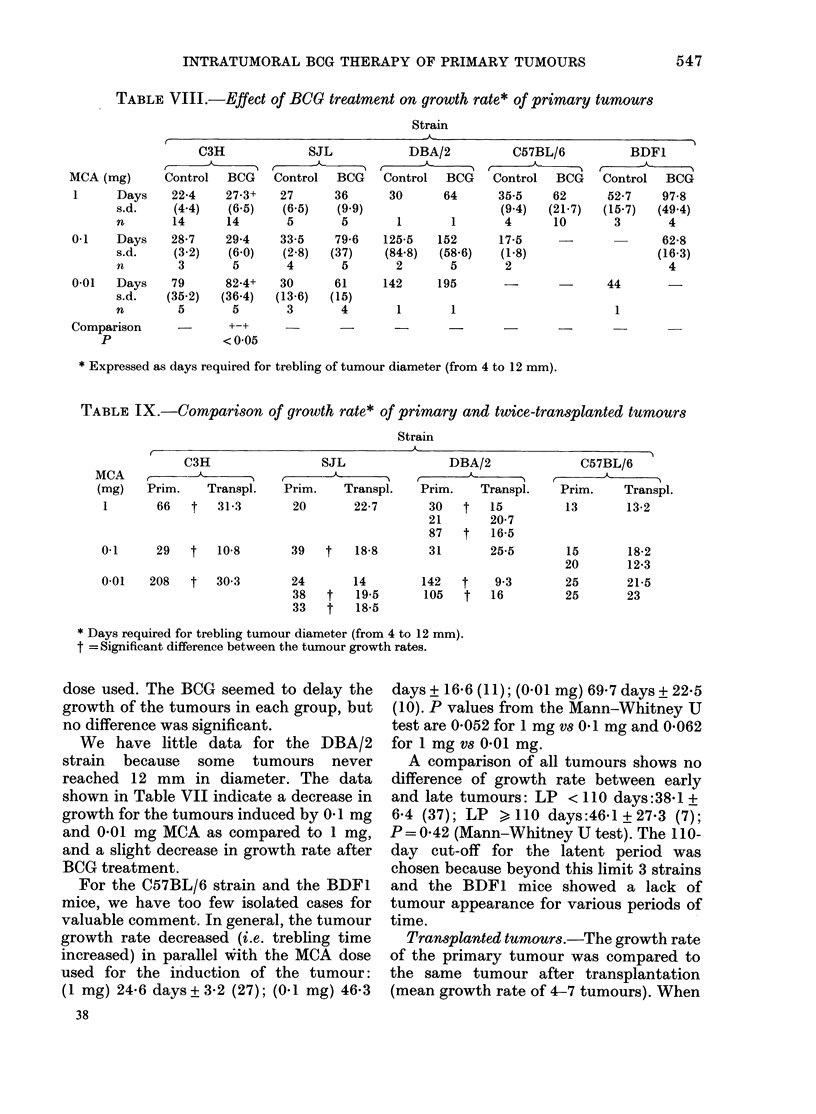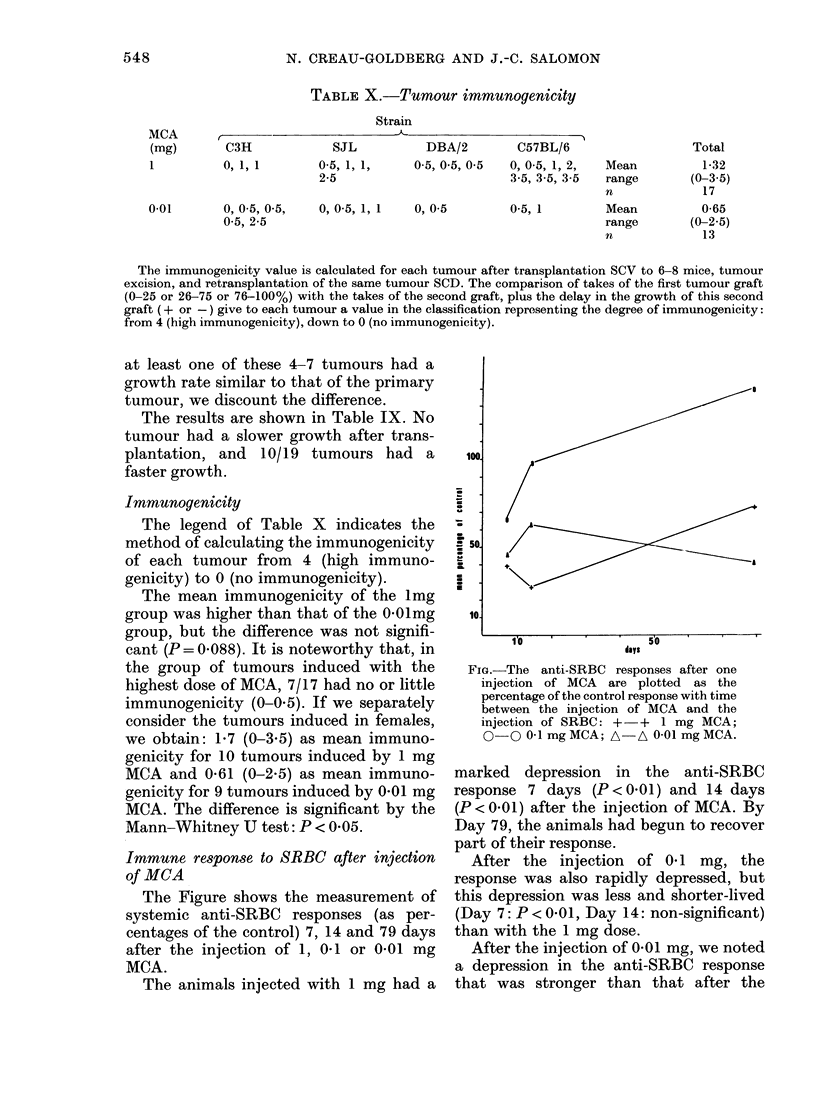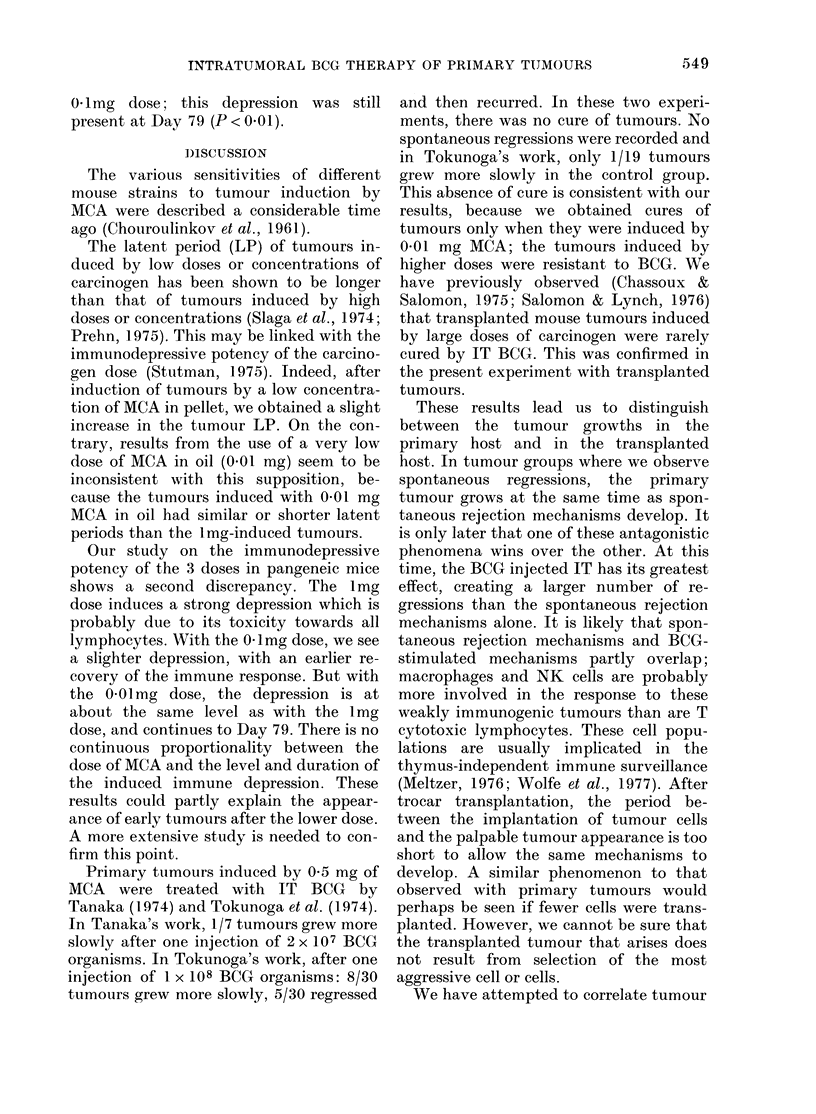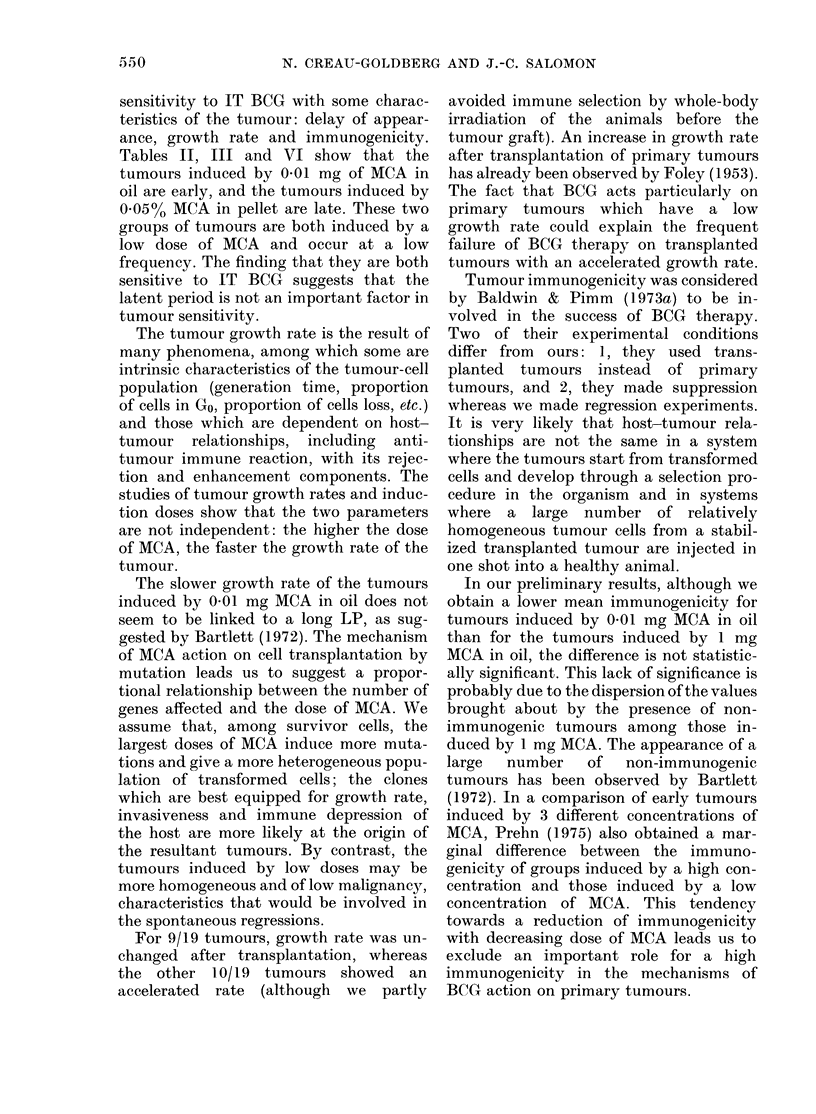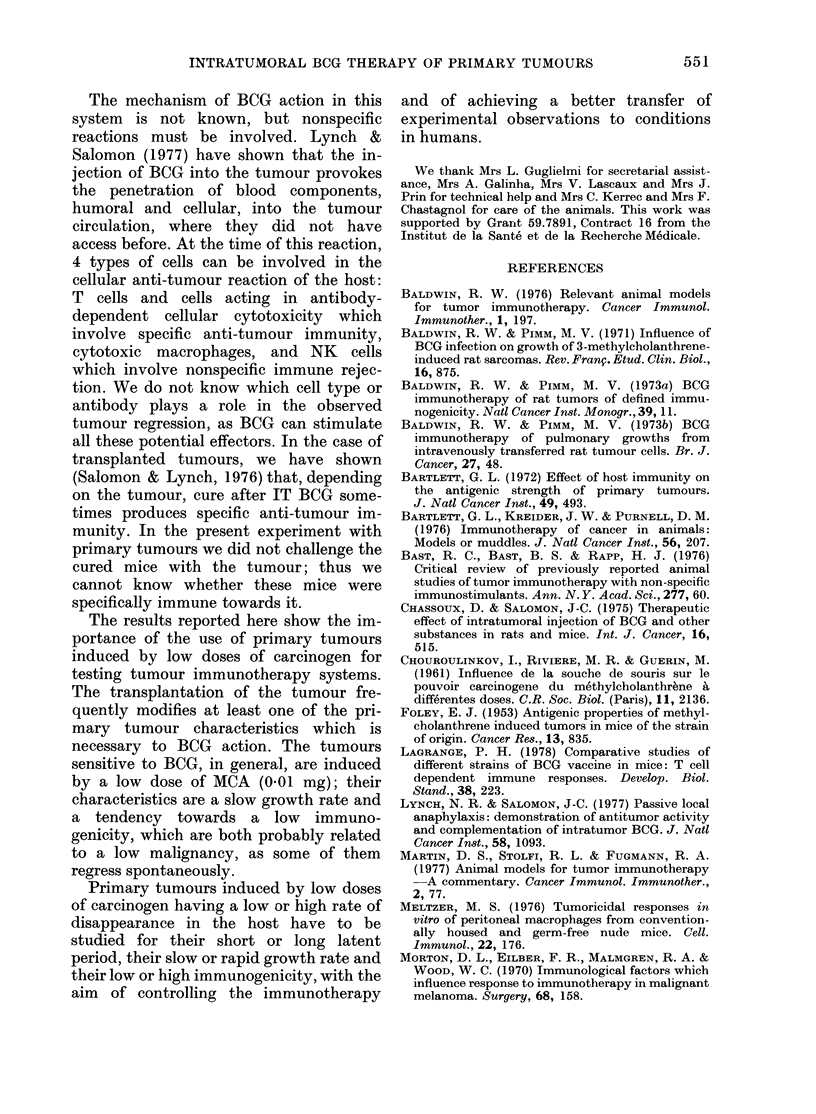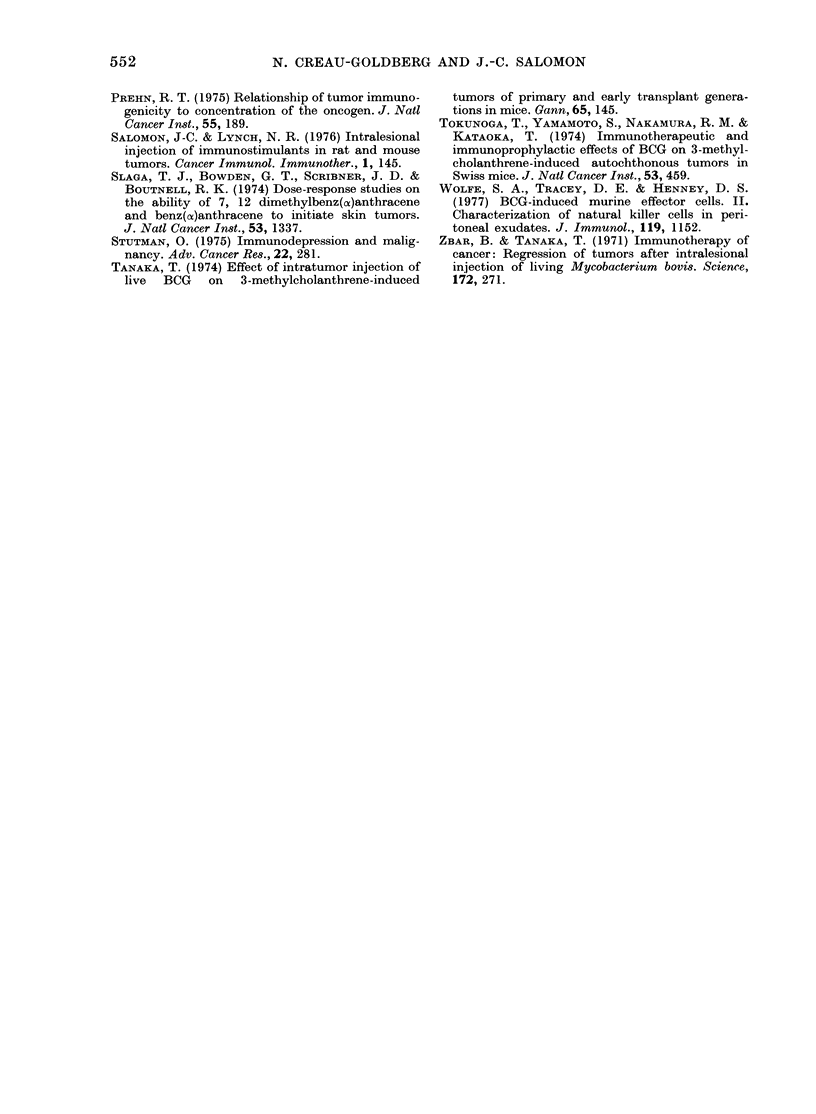# Immunotherapy of primary methylcholanthrene-induced mouse tumours by intratumoral BCG.

**DOI:** 10.1038/bjc.1980.96

**Published:** 1980-04

**Authors:** N. Creau-Goldberg, J. C. Salomon

## Abstract

Tumours were induced s.c. in C3H/uip, SJL/uip, DBA/2 uip, C57BL/6 uip and BDF1 mice by different doses of methylcholanthrene (MCA) diluted in oil: 1 mg, 0.1 mg and 0.01 mg. In each mouse strain, tumour frequency showed a different decreasing pattern in relation to the decreasing dose of MCA. Tumour latent period (LP) increased between the 1mg and 0.1mg doses of MCA, but the 0.01mg dose induced tumours with a similar or shorter LP than those tumours induced by 1 mg. Half of the tumours were treated with two injections of intratumoral (IT) BCG. The strains of mice differed in their sensitivity to this treatment, but only tumours induced by 0.01 mg MCA were sensitive to IT BCG. The induction of tumours by MCA pellets gave similar results. After transplantation of the untreated tumours, very few were cured by BCG treatment. Analysis of the role of tumour LP, growth rate and immunogenicity favours a slow growth rate as the most important characteristic for BCG sensitivity of the primary tumour. The tumours induced by 0.01 mg MCA were less immunogenic than those induced by 1 mg MCA, but the difference was not significant. This finding permits us to exclude an important role for tumour immunogenicity in the sensitivity of the primary tumour to BCB.


*Br. J. Cancer* (1980) **41**, 541

### IMMUNOTHERAPY OF PRIMARY METHYLCHOLANTHRENE-INDUCED MOUSE TUMOURS BY INTRATUMORAL BCG

N. CREAU-GOLDBERG* AND J.-C. SALOMON†

*From the Institut de Recherches Scientifiques sur le Cancer, CRNS, Villejuif, France*

Received 24 July 1979 Accepted 21 December 1979

**Summary.—Tumours were induced s.c. in C3H/uip, SJL/uip, DBA/2 uip, C57BL/6 uip and BDF1 mice by different doses of methylcholanthrene (MCA) diluted in oil: 1 mg, 0·1 mg and 0·01 mg. In each mouse strain, tumour frequency showed a different decreasing pattern in relation to the decreasing dose of MCA. Tumour latent period (LP) increased between the 1mg and 0·1mg doses of MCA, but the 0·01mg dose induced tumours with a similar or shorter LP than those tumours induced by 1 mg. Half of the tumours were treated with two injections of intratumoral (IT) BCG. The strains of mice differed in their sensitivity to this treatment, but only tumours induced by 0·01 mg MCA were sensitive to IT BCG. The induction of tumours by MCA pellets gave similar results. After transplantation of the untreated tumours, very few were cured by BCG treatment. Analysis of the role of tumour LP, growth rate and immunogenicity favours a slow growth rate as the most important characteristic for BCG sensitivity of the primary tumour. The tumours induced by 0·01 mg MCA were less immunogenic than those induced by 1 mg MCA, but the difference was not significant. This finding permits us to exclude an important role for tumour immunogenicity in the sensitivity of the primary tumour to BCG.**

AS RECENTLY CLAIMED by Baldwin (1976), Bartlett *et al.* (1976) and Martin *et al.* (1977) the results of tumour immunotherapy assays with transplanted tumours induced by large doses of carcinogen are not easily applied to human tumours.

"Spontaneous" tumours are not frequent enough in animals to allow the study of a particular treatment in many animals over a short time. A few animal strains have a high frequency of "spontaneous" tumours, but they have been selected for this character and do not represent a natural situation.

We attempted to avoid some of these problems by testing a tumour-immunotherapy system on primary tumours induced by various doses of carcinogen in different strains of mice. The immunotherapy used, injection of BCG into the tumours, was first described for the treatment of human melanoma (Morton *et al.*, 1970) transplanted guinea-pig tumour (Zbar & Tanaka, 1971) and transplanted rat tumours (Baldwin & Pimm, 1971; Chassoux & Salomon, 1975). Indeed this mode of treatment contains two of the important conditions for successful tumour immunotherapy compiled by Bast *et al.* (1976): a small tumour burden and direct contract between BCG and tumour cells. The BCG strain from the Pasteur Institute has been shown to be one of the most effective for tumour immunotherapy (Lagrange, 1978).

Some tumour characteristics have been studied to define more precisely the conditions of successful immunotherapy. Baldwin & Pimm (1973*a*, *b*) studied the role of tumour immunogenicity in the success of BCG treatment. Their work shows that the growth of several non-

* Fellow of the Ligue Nationale Française contre le Cancer. Present address: Hôpital des Enfants Malades, 149 rue de Sèvres, Paris, France.

‡ To whom correspondence should be sent.

immunogenic transplanted tumours is not suppressed by BCG (Baldwin & Pimm, 1973*a*) but that i.v. injection of BCG may inhibit the growth of pulmonary metastases of a weakly immunogenic tumour (Baldwin & Pimm, 1973*b*).

As far as we know, no systematic studies have been done on other tumour characteristics. Tokunoga *et al.* (1974) have shown that sensitivity to intratumoral (IT) BCG (regression plus recurrence of delay in growth) of primary tumours induced by 0·5 mg MCA was positively related to a short latent period (LP). Bartlett (1972) has also demonstrated an inverse correlation between the LP of the primary tumour and its growth rate. In the present study, the LP, growth rate and immunogenicity of primary tumours were examined in relation to the success or failure of therapy by IT BCG.

### MATERIALS AND METHODS

*Animals.*—Male and female C3H/uip, SJL/uip, DBA/2 uip, C57BL/6 uip and BDF1 mice, 2 months old, were obtained from the breeding facilities of our Institute. The pangeneic mice, 2 months old, were obtained from a random cross of mice of different strains; they were used at the 5th generation of random breeding.

*Tumours.*—Fibrosarcomas were induced by s.c. injection of 0·1 ml of olive oil containing 1 mg, 0·1 mg or 0·01 mg of MCA (Eastman Kodak). In a second experiment, this mode of induction was compared with the s.c. implantation of an MCA pellet (Prehn, 1975) containing 5% (0·2 mg), 0·5% (0·02 mg) or 0·05% (0·002 mg) MCA.

*Latent period* (LP).—Animals were examined for tumour appearance during one year. LP was recorded when the tumour diameter was 4 mm.

*Treatment of tumours.*—On Day 0 (day of MCA injection) mice were randomly designated to be treated or not treated by BCG when a tumour appeared.

When a primary tumour diameter reached 4–9 mm, the animal was given 2 IT injections of 1 mg BCG each (fresh BCG or immuno BCG of the Pasteur Institute) one week apart. The same treatment was applied to transplanted tumours (Chassoux & Salomon, 1975).

For the *C. parvum* treatment of tumours, the same schedule as for BCG was used. The only change was the dose used for each of the 2 injections: $10^9$ (0·07 mg) of heat-inactivated *C. parvum* (No. 4182 strain of the Pasteur Institute, provided by R. Ducluzeau, CRNS, Jouy-en-Josas, France).

The mice were considered as cured when no tumour was present at least one year after the injection of MCA, after a tumour had appeared at that site. When a node was still detected at the old tumour site, a histological study was done to exclude the possibility of an active tumour centre.

*Transplantation.*—Untreated tumours were excised when their diameters were 10–12 mm. At this size, the tumour mass was free of necrosis. Fragments of the tumour were frozen at −80°C in Hanks' medium containing 10% glycerol. These fragments were rapidly thawed and transplanted to animals that had been whole-body irradiated with 400 rad X-rays 24 h previously. Tumours that developed were then transplanted to different, untreated animals for the study of BCG immunotherapy or of tumour immunogenicity.

*Measurement of growth rate.*—For both primary and transplanted tumours, the growth rate was defined as the number of days necessary for the tumour to treble its diameter (4 to 12 mm). During this period the tumour growth is generally linear.

*Measurement of immunogenicity.* — The immunogenicity of the tumour was examined for untreated tumours induced by 1 mg or 0·01 mg MCA. Each tumour was transplanted s.c. on the abdomens (SCV) of 6–8 mice as a fragment of about 1 $mm^3$. When the tumour diameter reached 10–12 mm, the tumour was excised. Two weeks after the excision, a fragment of the same tumour was transplanted s.c. on to the backs (SCD) of these mice.

A recurrence at the first implantation site excluded the animal from the record of takes. The take and growth rates of the second tumour grafts indicate, when compared with those of the first tumour grafts, the degree of immunization of the animals. Each tumour was classified for immunogenicity (4 to 0) from the plotted results so that the different groups could be compared.

*Immune response to sheep red blood cells.*—At Day 0, $2 \times 10^8$ SRBC (Pasteur Institute) in 0·2 ml of phosphate-buffered saline were injected i.p. into each pangeneic mouse. At Day 5, the number of direct plaque-forming cells was determined for the spleen of each animal by Jerne's method.

### RESULTS

### *Tumour frequency*

Table I shows the various sensitivities of the different strains of mice to the induction of tumours by MCA. The 1mg dose gave the most tumours in all mouse strains. We can distinguish the strains by the different patterns of decrease in tumour frequency as a function of decrease in MCA dose. For instance, the SJL strain still had a high frequency of tumours at 0·01 mg MCA (13/19) and the C57BL/6 already had a low tumour frequency at the 0·1mg dose (2/15).

The strains in increasing order of sensitivity to MCA induction were: C57BL/6, C3H, DBA/2 and SJL. The pattern of decreasing tumour frequency among the F1 hybrids (C57BL/6 x DBA/2) was similar to that of the C57BL/6 strain, but with a higher frequency for the 2 lower doses of MCA.

### *Latent period* (LP)

Tumour appearance was recorded separately for male or female mice because of significant sex differences in the LP of tumours induced by 1 mg MCA (C3H and SJL).

Tables II and III show that, in general, the LP of the tumour increased between the 1mg and 0·1mg doses (SJL ♀, $P < 0{\cdot}05$; C3H ♂, $P < 0{\cdot}01$). The tumours induced with 0·01 mg MCA had an LP similar to or shorter than (C3H ♀, $P < 0{\cdot}05$; BDF1 ♀, $P < 0{\cdot}01$) those induced by 1 mg. *P* values were determined by the Mann–Whitney U test.

### *BCG treatment of the primary tumours*

No cure was seen for the 1mg MCA-induced tumours, and only one cure for those induced by 0·1 mg (Table IV).

TABLE I.—*Tumour frequency after injection of* 1, 0·1 *or* 0·01 *mg of MCA.* P *based on* $\chi^2$ *test after Yates' correction for small samples*

| MCA (mg) | Strain: C3H | SJL | DBA/2 | C57BL/6 | BDF1 | Comparison | *P** |
|---|---|---|---|---|---|---|---|
| 1 | $29/30^{x0}$ | 13/14 | 5/8 | $28/30^{x0}$ | $11/14^{x0}$ | DBA/2-C3H | < 0·05 |
| 0·1 | $9/14^{x}$ | 12/13 | $11/15^{x}$ | $2/15^{x}$ | $11/29^{x}$ | C57BL/6-C3H, SJL, DBA/2<br>BDF1-SJL, DBA/S | < 0·01 |
| 0·01 | $12/29^{0}$ | 13/19 | $11/26^{x}$ | $3/30^{0}$ | $6/15^{0}$ | C57BL/6-C3H, SJL, DBA/2<br>C57BL/6-BDF1, C3H-SJL | < 0·01<br>< 0·05 |
| Comparison | x–x and 0–0 | — | x–x | x–x and 0–0 | x–x 0–0 | | |
| *P* | < 0·01 | | < 0·05 | < 0·01 | < 0·01 < 0·05 | | |

TABLE II.—*Tumour latent period (in days) measured to when the tumour is about* 4 *mm in diameter. LPs of tumours that regressed spontaneously are excluded*

| MCA (mg) | | Strain: C3H ♀ | C3H ♂ | SJL ♀ | SJL ♂ |
|---|---|---|---|---|---|
| 1 | Mean | 97 | 58·3 | 63·4 | 74·5 |
| | Range | (79–271) | (53–66) | (43–140) | (67–77) |
| | *n* | 15 | 14 | 5 | 8 |
| 0·1 | Mean | — | 98·2 | 90·7 | — |
| | Range | | (69–102) | (63–145) | |
| | *n* | | 9 | 12 | |
| 0·01 | Mean | 99·7 | — | 81 | 83·4 |
| | Range | (56–265) | | (52–139) | (70–98) |
| | *n* | 12 | | 5 | 8 |

TABLE III.—*Tumour latent period (in days) measured to when the tumour is about 4 mm in diameter. LPs of tumours that regressed spontaneously are excluded*

| MCA (mg) | | Strain: DBA/2 ♀ | DBA/2 ♂ | C57BL/6 ♀ | C57BL/6 ♂ | BDF1 ♀ |
|---|---|---|---|---|---|---|
| 1 | Mean | — | 102·4 | 106·5 | — | 77·7 |
| | Range | | (63–225) | (70–295) | | (65–108) |
| | *n* | | 5 | 28 | | 11 |
| 0·1 | Mean | 139·9 | — | 198·5 | — | 89·1 |
| | Range | (70–271) | | (141–256) | | (49–138) |
| | *n* | 11 | | 2 | | 11 |
| 0·01 | Mean | 99·1 | — | 63 | 109 | 64·2 |
| | Range | (48–161) | | | (78–141) | (61–72) |
| | *n* | 11 | | 1 | 2 | 6 |

TABLE IV.—*Cure after BCG treatment of primary tumours. The primary tumours were treated when their diameter was 4–9 mm by 2 injections of 1 mg of BCG one week apart*

| MCA (mg) | | Strain: C3H | SJL | DBA/2 | C57BL/6 | BDF1 | Total |
|---|---|---|---|---|---|---|---|
| 1 | BCG | 0/11 | 0/6 | 0/1 | 0/10 | 0/4† | 0/32 |
| | Control | 0/29 | 0/7 | 0/4 | 0/15 | 0/7 | 0/62 |
| 0·1 | BCG | 0/5 | 0/6 | 0/5† | — | 1/7 | 1/23 |
| | Control | 0/15 | 0/7 | 0/4 | 0/2 | 0/3 | 0/31 |
| 0·01 | BCG | 2/5 | 0/6 | 7/8† | — | 3/3† | 12/22 |
| | Control | 0/6 | 0/5 | 5/8 | 0/3 | 1/4 | 6/23 |
| Comparison | | — | — | †—† | — | †—† | |
| *P* | | | | 0·009 | | 0·056 | |

There were many cures for the 0·01 mg MCA-induced tumours, but the sensitivity of the tumour to IT BCG varied between the strains of mice. The SJL strain was the most resistant and the DBA/2 strain the most sensitive of those studied. In the group of 0·01 mg MCA-induced tumours we also observed spontaneous regressions: 6/23 tumours regressed spontaneously compared with 12/22 tumours that regressed after IT BCG ($\chi^2 = 3{\cdot}79$, $P \sim 0{\cdot}05$).

A comparison of survival among treated and untreated mice was not made, because the untreated tumours were excised and frozen for the later study of BCG immunotherapy on transplanted tumours.

*BCG treatment of the transplanted tumours*

BCG cured very few transplanted tumours (Table V): 2/6 SJL tumours transplanted from a 1mg-induced tumour (1/5 spontaneous regression was recorded in the controls) and 1/5 C57BL/6 tumours transplanted from a 0·01mg-induced tumour.

In progressors, the survival time after treatment with IT BCG was compared by Student's *t* test with survival time of untreated animals, and was similar in the 2 groups. BCG treatment did not affect the tumour growth rate either way.

*Comparison of the modes of tumour induction: oil and pellet*

Only the C3H strain of mice was used in this experiment.

As shown in Table VI, the 2 modes of induction did not differ in the frequency of tumours produced, or in the sensitivity of such tumours to BCG or *C. parvum*

TABLE V.—*Survival time (days + s.d.) and number of cured animals after BCG treatment of the tumours at the second transplantation. The tumours were treated when they were 4 mm in diameter by 2 injections of 1 mg BCG one week apart. LP refers to the primary tumour. Figures for control animals are not given*

| | | MCA (mg) | | |
|---|---|---|---|---|
| Strain | | 1 | 0·1 | 0·01 |
| C3H | LP (days) | 60 | 69 | 56 |
| | control (days) | 59·5 (5) | 39·8 (2·4) | 62·7 (5) |
| | BCG (days) | 67·4 (3) | 39 (2·3) | 59 (2) |
| | cure/treat. | 0/6 | 0/6 | 0/6 |
| SJL | LP | 140 | 70 | 76 |
| | control | 43·8 (7·2) | 34·2 (4·4) | 49·6 (3·5) |
| | BCG | 53·5 (4) | 30·4 (4·1) | 38·5 (6·7) |
| | cure/treat. | 2/6 | 0/5 | 0/6 |
| | LP | — | — | 52 |
| | control | | | 43·8 (3·6) |
| | BCG | | | 46·2 (2·3) |
| | cure/treat. | | | 0/6 |
| | LP | — | — | 139 |
| | control | | | 36 (2·3) |
| | BCG | | | 32·2 (5·4) |
| | cure/treat. | | | 0/6 |
| | LP | — | — | 71 |
| | control | | | 72 (8·9) |
| | BCG | | | 64 (9·4) |
| | cure/treat. | | | 0/5 |
| DBA/2 | LP | 63 | 151 | 48 |
| | control | 31·3 (1·7) | 46 (0) | 27 (1·3) |
| | BCG | 30·9 (1·7) | 52·4 (3·3) | 28·7 (1) |
| | cure/treat. | 0/8 | 0/8 | 0/6 |
| | LP | 100 | — | 161 |
| | control | 40·5 (4·2) | | 41·1 (1·7) |
| | BCG | 40·3 (1·9) | | 42 (1·1) |
| | cure/treat. | 0/6 | | 0/8 |
| | LP | 61 | — | — |
| | control | 29·2 (1·2) | | |
| | BCG | 30·3 (1·5) | | |
| | cure/treat. | 0/6 | | |
| C57BL/6 | LP | 82 | 141 | 141 |
| | control | 42·6 (4·8) | 46·3 (3·8) | 58·3 (3·9) |
| | BCG | 36 (3) | 41 (2·5) | 53·3 (2·7) |
| | cure/treat. | 0/5 | 0/6 | 0/3 |
| | LP | — | 256 | 77 |
| | control | | 40 (4) | 64·2 (6·7) |
| | BCG | | 40 (2·1) | 49·3 (2·3) |
| | cure/treat. | | 0/6 | 1/5 |

treatment. The effect of the treatment was similar to that seen in the first experiment: cures were obtained only for tumours induced by the lower doses or the lower concentrations of MCA.

The two modes differed in the LP of the tumours that developed: there was an increase in LP parallel to the decrease in concentration of MCA in pellet-induced tumours. However, the differences were not significant.

*Sensitivity to BCG treatment of the primary tumours in relation to latent period*

Table VII shows the results obtained after the classification of tumours (independently of the dose of MCA used for their induction) for their sensitivity to BCG treatment: (−) resistance, (±) regression and recurrence, (+) cure. In DBA/2 and BDF1 mice, the early tumours were more sensitive to BCG treatment than the late tumours. The significance of

TABLE VI.—*Comparison of the induction of tumours by MCA in oil or in Millipore paraffin pellets, in the C3H strain, on the basis of frequency, mean latent period and sensitivity to BCG or* C. parvum *treatment of tumours induced by the 2 modes of induction. No significant differences in cure rates ($\chi^2$ test). The only significant difference in LP (Mann–Whitney U test) is between* 1 *and* 0·1 *mg MCA* (P < 0·01)

| | MCA | Tumour frequency | LP in days (s.d.) | Cure rate BCG | Cure rate *C. parvum* |
|---|---|---|---|---|---|
| Oil | 1 mg | 10/10 | 70·9 (6·1) | 0/5 | 0/5 |
| | 0·1 mg | 15/15 | 88·8 (7·1) | 0/8 | 1/7 |
| | 0·01 mg | 11/20 | 68·1 (3·6) | 1/6 | 2/5 |
| Pellet | 5% | 10/10 | 90·8 (7·0) | 0/5 | 0/5 |
| | 0·5% | 14/15 | 107·1 (10·2) | 0/5 | 0/5 |
| | 0·05% | 12/20 | 102 (9·1) | 1/5 | 2/6 |
| | 0% | 0/10 | — | — | — |

TABLE VII.—*Comparison between latent period and sensitivity to BCG treatment of primary tumours*

| Sensitivity to BCG treatment* | | Strain C3H (1+0·1+0·01)† | SJL (1+0·1+0·01) | DBA/2 (1+0·1+0·01) | C57BL/6 (1) | BDF1 (1+0·1+0·01) |
|---|---|---|---|---|---|---|
| + | Mean LP | 74·5 | — | 92ˣ | — | 61·8ˣ |
| | s.d. | (2·5) | | (8·2) | | (1·7) |
| | *n* | 2 | | 7 | | 4 |
| ± | Mean LP | 72 | 79·8 | 132 | — | 72 |
| | s.d. | (11·6) | (6·9) | (48) | | |
| | *n* | 3 | 5 | 2 | | 1 |
| − | Mean LP | 77·4 | 76·8 | 139·6ˣ | 102·1 | 97·5ˣ |
| | s.d. | (3·8) | (7·6) | (23) | (8·5) | (10·1) |
| | *n* | 16 | 12 | 5 | 9 | 10 |
| Comparison | | | | x–x | | x–x |
| *P*‡ | | — | — | 0·053 | — | < 0·05 |

* +, cure; ±, regression and recurrence; −, resistance.
† mg doses of MCA used to induce the primary tumours.
‡ *P* based on the Mann–Whitney U test.
LP values for tumours which regressed spontaneously are: 91·2 (8·7) for 5 DBA/2 tumours and 83 for the only BDF1 tumour.

this difference is due mostly to the earliness of the tumours induced by 0·01 mg MCA in oil.

*Tumour growth rates*

*Primary tumours* (Table VIII).—We calculated the growth rates for untreated (control) and treated (BCG) tumours of each group. Some tumours could not be recorded in this table because of the limits chosen.

For the C3H strain, the tumour growth rate was similar for treated and untreated tumours. All tumours induced by the lowest dose of MCA had a slow growth rate.

For the SJL strain, the tumour growth rate was nearly the same for every MCA

TABLE VIII.—*Effect of BCG treatment on growth rate* of primary tumours*

| MCA (mg) | | C3H Control | C3H BCG | SJL Control | SJL BCG | DBA/2 Control | DBA/2 BCG | C57BL/6 Control | C57BL/6 BCG | BDF1 Control | BDF1 BCG |
|---|---|---|---|---|---|---|---|---|---|---|---|
| 1 | Days | 22·4 | 27·3+ | 27 | 36 | 30 | 64 | 35·5 | 62 | 52·7 | 97·8 |
| | s.d. | (4·4) | (6·5) | (6·5) | (9·9) | | | (9·4) | (21·7) | (15·7) | (49·4) |
| | *n* | 14 | 14 | 5 | 5 | 1 | 1 | 4 | 10 | 3 | 4 |
| 0·1 | Days | 28·7 | 29·4 | 33·5 | 79·6 | 125·5 | 152 | 17·5 | — | — | 62·8 |
| | s.d. | (3·2) | (6·0) | (2·8) | (37) | (84·8) | (58·6) | (1·8) | | | (16·3) |
| | *n* | 3 | 5 | 4 | 5 | 2 | 5 | 2 | | | 4 |
| 0·01 | Days | 79 | 82·4+ | 30 | 61 | 142 | 195 | — | — | 44 | — |
| | s.d. | (35·2) | (36·4) | (13·6) | (15) | | | | | | |
| | *n* | 5 | 5 | 3 | 4 | 1 | 1 | | | 1 | |
| Comparison | | — | +–+ | — | — | — | — | — | — | — | — |
| *P* | | | < 0·05 | | | | | | | | |

* Expressed as days required for trebling of tumour diameter (from 4 to 12 mm).

TABLE IX.—*Comparison of growth rate* of primary and twice-transplanted tumours*

| MCA (mg) | C3H Prim. | | C3H Transpl. | SJL Prim. | | SJL Transpl. | DBA/2 Prim. | | DBA/2 Transpl. | C57BL/6 Prim. | C57BL/6 Transpl. |
|---|---|---|---|---|---|---|---|---|---|---|---|
| 1 | 66 | † | 31·3 | 20 | | 22·7 | 30 | † | 15 | 13 | 13·2 |
| | | | | | | | 21 | | 20·7 | | |
| | | | | | | | 87 | † | 16·5 | | |
| 0·1 | 29 | † | 10·8 | 39 | † | 18·8 | 31 | | 25·5 | 15 | 18·2 |
| | | | | | | | | | | 20 | 12·3 |
| 0·01 | 208 | † | 30·3 | 24 | | 14 | 142 | † | 9·3 | 25 | 21·5 |
| | | | | 38 | † | 19·5 | 105 | † | 16 | 25 | 23 |
| | | | | 33 | † | 18·5 | | | | | |

* Days required for trebling tumour diameter (from 4 to 12 mm).
† = Significant difference between the tumour growth rates.

dose used. The BCG seemed to delay the growth of the tumours in each group, but no difference was significant.

We have little data for the DBA/2 strain because some tumours never reached 12 mm in diameter. The data shown in Table VII indicate a decrease in growth for the tumours induced by 0·1 mg and 0·01 mg MCA as compared to 1 mg, and a slight decrease in growth rate after BCG treatment.

For the C57BL/6 strain and the BDF1 mice, we have too few isolated cases for valuable comment. In general, the tumour growth rate decreased (*i.e.* trebling time increased) in parallel with the MCA dose used for the induction of the tumour: (1 mg) 24·6 days ± 3·2 (27); (0·1 mg) 46·3 days ± 16·6 (11); (0·01 mg) 69·7 days ± 22·5 (10). *P* values from the Mann–Whitney U test are 0·052 for 1 mg *vs* 0·1 mg and 0·062 for 1 mg *vs* 0·01 mg.

A comparison of all tumours shows no difference of growth rate between early and late tumours: LP < 110 days:38·1 ± 6·4 (37); LP ⩾ 110 days:46·1 ± 27·3 (7); *P* = 0·42 (Mann–Whitney U test). The 110-day cut-off for the latent period was chosen because beyond this limit 3 strains and the BDF1 mice showed a lack of tumour appearance for various periods of time.

*Transplanted tumours*.—The growth rate of the primary tumour was compared to the same tumour after transplantation (mean growth rate of 4–7 tumours). When

TABLE X.—*Tumour immunogenicity*

| MCA (mg) | Strain: C3H | SJL | DBA/2 | C57BL/6 | | Total |
|---|---|---|---|---|---|---|
| 1 | 0, 1, 1 | 0·5, 1, 1, 2·5 | 0·5, 0·5, 0·5 | 0, 0·5, 1, 2, 3·5, 3·5, 3·5 | Mean | 1·32 |
| | | | | | range | (0–3·5) |
| | | | | | *n* | 17 |
| 0·01 | 0, 0·5, 0·5, 0·5, 2·5 | 0, 0·5, 1, 1 | 0, 0·5 | 0·5, 1 | Mean | 0·65 |
| | | | | | range | (0–2·5) |
| | | | | | *n* | 13 |

The immunogenicity value is calculated for each tumour after transplantation SCV to 6–8 mice, tumour excision, and retransplantation of the same tumour SCD. The comparison of takes of the first tumour graft (0–25 or 26–75 or 76–100%) with the takes of the second graft, plus the delay in the growth of this second graft (+ or −) give to each tumour a value in the classification representing the degree of immunogenicity: from 4 (high immunogenicity), down to 0 (no immunogenicity).

at least one of these 4–7 tumours had a growth rate similar to that of the primary tumour, we discount the difference.

The results are shown in Table IX. No tumour had a slower growth after transplantation, and 10/19 tumours had a faster growth.

*Immunogenicity*

The legend of Table X indicates the method of calculating the immunogenicity of each tumour from 4 (high immunogenicity) to 0 (no immunogenicity).

The mean immunogenicity of the 1mg group was higher than that of the 0·01mg group, but the difference was not significant ($P = 0{\cdot}088$). It is noteworthy that, in the group of tumours induced with the highest dose of MCA, 7/17 had no or little immunogenicity (0–0·5). If we separately consider the tumours induced in females, we obtain: 1·7 (0–3·5) as mean immunogenicity for 10 tumours induced by 1 mg MCA and 0·61 (0–2·5) as mean immunogenicity for 9 tumours induced by 0·01 mg MCA. The difference is significant by the Mann–Whitney U test: $P < 0{\cdot}05$.

*Immune response to SRBC after injection of MCA*

The Figure shows the measurement of systemic anti-SRBC responses (as percentages of the control) 7, 14 and 79 days after the injection of 1, 0·1 or 0·01 mg MCA.

The animals injected with 1 mg had a marked depression in the anti-SRBC response 7 days ($P < 0{\cdot}01$) and 14 days ($P < 0{\cdot}01$) after the injection of MCA. By Day 79, the animals had begun to recover part of their response.

FIG.—The anti-SRBC responses after one injection of MCA are plotted as the percentage of the control response with time between the injection of MCA and the injection of SRBC: +—+ 1 mg MCA; ○—○ 0·1 mg MCA; △—△ 0·01 mg MCA.

After the injection of 0·1 mg, the response was also rapidly depressed, but this depression was less and shorter-lived (Day 7: $P < 0{\cdot}01$, Day 14: non-significant) than with the 1 mg dose.

After the injection of 0·01 mg, we noted a depression in the anti-SRBC response that was stronger than that after the

0·1mg dose; this depression was still present at Day 79 ($P < 0.01$).

### DISCUSSION

The various sensitivities of different mouse strains to tumour induction by MCA were described a considerable time ago (Chouroulinkov *et al.*, 1961).

The latent period (LP) of tumours induced by low doses or concentrations of carcinogen has been shown to be longer than that of tumours induced by high doses or concentrations (Slaga *et al.*, 1974; Prehn, 1975). This may be linked with the immunodepressive potency of the carcinogen dose (Stutman, 1975). Indeed, after induction of tumours by a low concentration of MCA in pellet, we obtained a slight increase in the tumour LP. On the contrary, results from the use of a very low dose of MCA in oil (0·01 mg) seem to be inconsistent with this supposition, because the tumours induced with 0·01 mg MCA in oil had similar or shorter latent periods than the 1mg-induced tumours.

Our study on the immunodepressive potency of the 3 doses in pangeneic mice shows a second discrepancy. The 1mg dose induces a strong depression which is probably due to its toxicity towards all lymphocytes. With the 0·1mg dose, we see a slighter depression, with an earlier recovery of the immune response. But with the 0·01mg dose, the depression is at about the same level as with the 1mg dose, and continues to Day 79. There is no continuous proportionality between the dose of MCA and the level and duration of the induced immune depression. These results could partly explain the appearance of early tumours after the lower dose. A more extensive study is needed to confirm this point.

Primary tumours induced by 0·5 mg of MCA were treated with IT BCG by Tanaka (1974) and Tokunoga *et al.* (1974). In Tanaka's work, 1/7 tumours grew more slowly after one injection of $2 \times 10^7$ BCG organisms. In Tokunoga's work, after one injection of $1 \times 10^8$ BCG organisms: 8/30 tumours grew more slowly, 5/30 regressed and then recurred. In these two experiments, there was no cure of tumours. No spontaneous regressions were recorded and in Tokunoga's work, only 1/19 tumours grew more slowly in the control group. This absence of cure is consistent with our results, because we obtained cures of tumours only when they were induced by 0·01 mg MCA; the tumours induced by higher doses were resistant to BCG. We have previously observed (Chassoux & Salomon, 1975; Salomon & Lynch, 1976) that transplanted mouse tumours induced by large doses of carcinogen were rarely cured by IT BCG. This was confirmed in the present experiment with transplanted tumours.

These results lead us to distinguish between the tumour growths in the primary host and in the transplanted host. In tumour groups where we observe spontaneous regressions, the primary tumour grows at the same time as spontaneous rejection mechanisms develop. It is only later that one of these antagonistic phenomena wins over the other. At this time, the BCG injected IT has its greatest effect, creating a larger number of regressions than the spontaneous rejection mechanisms alone. It is likely that spontaneous rejection mechanisms and BCG-stimulated mechanisms partly overlap; macrophages and NK cells are probably more involved in the response to these weakly immunogenic tumours than are T cytotoxic lymphocytes. These cell populations are usually implicated in the thymus-independent immune surveillance (Meltzer, 1976; Wolfe *et al.*, 1977). After trocar transplantation, the period between the implantation of tumour cells and the palpable tumour appearance is too short to allow the same mechanisms to develop. A similar phenomenon to that observed with primary tumours would perhaps be seen if fewer cells were transplanted. However, we cannot be sure that the transplanted tumour that arises does not result from selection of the most aggressive cell or cells.

We have attempted to correlate tumour sensitivity to IT BCG with some characteristics of the tumour: delay of appearance, growth rate and immunogenicity. Tables II, III and VI show that the tumours induced by 0·01 mg of MCA in oil are early, and the tumours induced by 0·05% MCA in pellet are late. These two groups of tumours are both induced by a low dose of MCA and occur at a low frequency. The finding that they are both sensitive to IT BCG suggests that the latent period is not an important factor in tumour sensitivity.

The tumour growth rate is the result of many phenomena, among which some are intrinsic characteristics of the tumour-cell population (generation time, proportion of cells in $G_0$, proportion of cells loss, *etc.*) and those which are dependent on host–tumour relationships, including anti-tumour immune reaction, with its rejection and enhancement components. The studies of tumour growth rates and induction doses show that the two parameters are not independent: the higher the dose of MCA, the faster the growth rate of the tumour.

The slower growth rate of the tumours induced by 0·01 mg MCA in oil does not seem to be linked to a long LP, as suggested by Bartlett (1972). The mechanism of MCA action on cell transplantation by mutation leads us to suggest a proportional relationship between the number of genes affected and the dose of MCA. We assume that, among survivor cells, the largest doses of MCA induce more mutations and give a more heterogeneous population of transformed cells; the clones which are best equipped for growth rate, invasiveness and immune depression of the host are more likely at the origin of the resultant tumours. By contrast, the tumours induced by low doses may be more homogeneous and of low malignancy, characteristics that would be involved in the spontaneous regressions.

For 9/19 tumours, growth rate was unchanged after transplantation, whereas the other 10/19 tumours showed an accelerated rate (although we partly avoided immune selection by whole-body irradiation of the animals before the tumour graft). An increase in growth rate after transplantation of primary tumours has already been observed by Foley (1953). The fact that BCG acts particularly on primary tumours which have a low growth rate could explain the frequent failure of BCG therapy on transplanted tumours with an accelerated growth rate.

Tumour immunogenicity was considered by Baldwin & Pimm (1973*a*) to be involved in the success of BCG therapy. Two of their experimental conditions differ from ours: 1, they used transplanted tumours instead of primary tumours, and 2, they made suppression whereas we made regression experiments. It is very likely that host–tumour relationships are not the same in a system where the tumours start from transformed cells and develop through a selection procedure in the organism and in systems where a large number of relatively homogeneous tumour cells from a stabilized transplanted tumour are injected in one shot into a healthy animal.

In our preliminary results, although we obtain a lower mean immunogenicity for tumours induced by 0·01 mg MCA in oil than for the tumours induced by 1 mg MCA in oil, the difference is not statistically significant. This lack of significance is probably due to the dispersion of the values brought about by the presence of non-immunogenic tumours among those induced by 1 mg MCA. The appearance of a large number of non-immunogenic tumours has been observed by Bartlett (1972). In a comparison of early tumours induced by 3 different concentrations of MCA, Prehn (1975) also obtained a marginal difference between the immunogenicity of groups induced by a high concentration and those induced by a low concentration of MCA. This tendency towards a reduction of immunogenicity with decreasing dose of MCA leads us to exclude an important role for a high immunogenicity in the mechanisms of BCG action on primary tumours.

The mechanism of BCG action in this system is not known, but nonspecific reactions must be involved. Lynch & Salomon (1977) have shown that the injection of BCG into the tumour provokes the penetration of blood components, humoral and cellular, into the tumour circulation, where they did not have access before. At the time of this reaction, 4 types of cells can be involved in the cellular anti-tumour reaction of the host: T cells and cells acting in antibody-dependent cellular cytotoxicity which involve specific anti-tumour immunity, cytotoxic macrophages, and NK cells which involve nonspecific immune rejection. We do not know which cell type or antibody plays a role in the observed tumour regression, as BCG can stimulate all these potential effectors. In the case of transplanted tumours, we have shown (Salomon & Lynch, 1976) that, depending on the tumour, cure after IT BCG sometimes produces specific anti-tumour immunity. In the present experiment with primary tumours we did not challenge the cured mice with the tumour; thus we cannot know whether these mice were specifically immune towards it.

The results reported here show the importance of the use of primary tumours induced by low doses of carcinogen for testing tumour immunotherapy systems. The transplantation of the tumour frequently modifies at least one of the primary tumour characteristics which is necessary to BCG action. The tumours sensitive to BCG, in general, are induced by a low dose of MCA (0·01 mg); their characteristics are a slow growth rate and a tendency towards a low immunogenicity, which are both probably related to a low malignancy, as some of them regress spontaneously.

Primary tumours induced by low doses of carcinogen having a low or high rate of disappearance in the host have to be studied for their short or long latent period, their slow or rapid growth rate and their low or high immunogenicity, with the aim of controlling the immunotherapy and of achieving a better transfer of experimental observations to conditions in humans.

We thank Mrs L. Guglielmi for secretarial assistance, Mrs A. Galinha, Mrs V. Lascaux and Mrs J. Prin for technical help and Mrs C. Kerrec and Mrs F. Chastagnol for care of the animals. This work was supported by Grant 59.7891, Contract 16 from the Institut de la Santé et de la Recherche Médicale.

### REFERENCES

BALDWIN, R. W. (1976) Relevant animal models for tumor immunotherapy. *Cancer Immunol. Immunother.*, **1**, 197.

BALDWIN, R. W. & PIMM, M. V. (1971) Influence of BCG infection on growth of 3-methylcholanthrene-induced rat sarcomas. *Rev. Franç. Etud. Clin. Biol.*, **16**, 875.

BALDWIN, R. W. & PIMM, M. V. (1973*a*) BCG immunotherapy of rat tumors of defined immunogenicity. *Natl Cancer Inst. Monogr.*, **39**, 11.

BALDWIN, R. W. & PIMM, M. V. (1973*b*) BCG immunotherapy of pulmonary growths from intravenously transferred rat tumour cells. *Br. J. Cancer*, **27**, 48.

BARTLETT, G. L. (1972) Effect of host immunity on the antigenic strength of primary tumours. *J. Natl Cancer Inst.*, **49**, 493.

BARTLETT, G. L., KREIDER, J. W. & PURNELL, D. M. (1976) Immunotherapy of cancer in animals: Models or muddles. *J. Natl Cancer Inst.*, **56**, 207.

BAST, R. C., BAST, B. S. & RAPP, H. J. (1976) Critical review of previously reported animal studies of tumor immunotherapy with non-specific immunostimulants. *Ann. N.Y. Acad. Sci.*, **277**, 60.

CHASSOUX, D. & SALOMON, J-C. (1975) Therapeutic effect of intratumoral injection of BCG and other substances in rats and mice. *Int. J. Cancer*, **16**, 515.

CHOUROULINKOV, I., RIVIERE, M. R. & GUERIN, M. (1961) Influence de la souche de souris sur le pouvoir carcinogene du méthylcholanthrène à differentes doses. *C.R. Soc. Biol.* (Paris), **11**, 2136.

FOLEY, E. J. (1953) Antigenic properties of methylcholanthrene induced tumors in mice of the strain of origin. *Cancer Res.*, **13**, 835.

LAGRANGE, P. H. (1978) Comparative studies of different strains of BCG vaccine in mice: T cell dependent immune responses. *Develop. Biol. Stand.*, **38**, 223.

LYNCH, N. R. & SALOMON, J-C. (1977) Passive local anaphylaxis: demonstration of antitumor activity and complementation of intratumor BCG. *J. Natl Cancer Inst.*, **58**, 1093.

MARTIN, D. S., STOLFI, R. L. & FUGMANN, R. A. (1977) Animal models for tumor immunotherapy —A commentary. *Cancer Immunol. Immunother.*, **2**, 77.

MELTZER, M. S. (1976) Tumoricidal responses *in vitro* of peritoneal macrophages from conventionally housed and germ-free nude mice. *Cell. Immunol.*, **22**, 176.

MORTON, D. L., EILBER, F. R., MALMGREN, R. A. & WOOD, W. C. (1970) Immunological factors which influence response to immunotherapy in malignant melanoma. *Surgery*, **68**, 158.

PREHN, R. T. (1975) Relationship of tumor immunogenicity to concentration of the oncogen. *J. Natl Cancer Inst.*, **55**, 189.

SALOMON, J-C. & LYNCH, N. R. (1976) Intralesional injection of immunostimulants in rat and mouse tumors. *Cancer Immunol. Immunother.*, **1**, 145.

SLAGA, T. J., BOWDEN, G. T., SCRIBNER, J. D. & BOUTNELL, R. K. (1974) Dose-response studies on the ability of 7, 12 dimethylbenz(α)anthracene and benz(α)anthracene to initiate skin tumors. *J. Natl Cancer Inst.*, **53**, 1337.

STUTMAN, O. (1975) Immunodepression and malignancy. *Adv. Cancer Res.*, **22**, 281.

TANAKA, T. (1974) Effect of intratumor injection of live BCG on 3-methylcholanthrene-induced tumors of primary and early transplant generations in mice. *Gann*, **65**, 145.

TOKUNOGA, T., YAMAMOTO, S., NAKAMURA, R. M. & KATAOKA, T. (1974) Immunotherapeutic and immunoprophylactic effects of BCG on 3-methylcholanthrene-induced autochthonous tumors in Swiss mice. *J. Natl Cancer Inst.*, **53**, 459.

WOLFE, S. A., TRACEY, D. E. & HENNEY, D. S. (1977) BCG-induced murine effector cells. II. Characterization of natural killer cells in peritoneal exudates. *J. Immunol.*, **119**, 1152.

ZBAR, B. & TANAKA, T. (1971) Immunotherapy of cancer: Regression of tumors after intralesional injection of living *Mycobacterium bovis*. *Science*, **172**, 271.